# Association of low fat mass with nontuberculous mycobacterial infection in patients with bronchiectasis

**DOI:** 10.1097/MD.0000000000025193

**Published:** 2021-04-09

**Authors:** Sung Yoon Lim, Yeon Joo Lee, Jong Sun Park, Young-Jae Cho, Ho Il Yoon, Choon-Taek Lee, Jae Ho Lee

**Affiliations:** Division of Pulmonary and Critical Care Medicine, Department of Internal Medicine, Seoul National University Bundang Hospital, Seongnam-si, Gyeonggi-do, Republic of Korea.

**Keywords:** bioelectrical impedance analysis, bronchiectasis, fat mass, nontuberculous mycobacterial infection, skeletal muscle mass

## Abstract

The incidence of pulmonary nontuberculous mycobacterial (NTM) infection is high in patients with underlying lung disease such as bronchiectasis. Although previous studies have reported many risk factors contributing to the development of NTM-lung disease (LD), only a few reports on the relationship of the characteristics of patients, such as body mass index (BMI), skeletal mass, and fat mass, with NTM-LD have been published. We aimed to investigate the association between these parameters and NTM-LD in patients with bronchiectasis.

A monocentric retrospective study in a university hospital was conducted over 4 years (2013–2016). Parameters including BMI, skeletal mass, and fat mass were measured using bioelectrical impedance analysis in noncystic fibrosis bronchiectasis patients. Patients were grouped by the presence or absence of NTM-LD, and the differences in BMI, skeletal mass, and fat mass between the 2 groups were compared. In the NTM-LD group, the progression of disease was also followed.

Two hundred forty-five patients with bronchiectasis were enrolled in the study. One hundred six subjects (48%) had NTM-LD. These patients with NTM-LD were predominantly female, and had a significantly lower body weight (58.20 ± 8.84 vs 54.16 ± 8.99, *P* < .001), BMI (22.67 ± 3.04 vs 21.20 ± 2.59 kg/m^2^, *P* < .001), fat mass (16.19 ± 6.60 vs 14.23 ± 5.79, *P* = .013), and fat mass index (FMI; 6.79 ± 2.70 vs 5.57 ± 2.27 kg/m^2^, *P* < .001). Multivariate regression analysis showed that both female sex and lower FMI but not skeletal muscle index were independent risk factors for NTM-LD after adjusting for age, bronchiectasis severity index, and BMI (odds ratio 3.86 (1.99–7.78); 0.72 (0.63–0.82), *P* < .001, respectively).

Our results suggest that lower FMI may contribute to susceptibility to NTM infection in patients with bronchiectasis, independent of age or its severity.

## 1 Introduction

The prevalence of nontuberculous mycobacterial (NTM) lung disease (LD) is increasing in many parts of the world and the burden now exceeds that of *Mycobacterium tuberculosis* in some countries.^[1]^ The incidence and NTM-related mortality have significantly increased in the last decades as well.^[2]^ Improved diagnostic methods and increased physician awareness also might have contributed to such an increase in NTM-LD. Additionally, it has been suggested that the significant increase of NTM-LD is caused by the accompanying growth of the aging population and increased comorbid lung diseases such as bronchiectasis, chronic obstructive pulmonary disease (COPD), lung cancer, and interstitial lung disease.^[3]^

Bronchiectasis is defined as an irreversible dilation of the bronchi, involving a vicious circle of transmural infection. It typically begins with airway inflammation almost always after an infectious process,^[4]^ leading to subsequent permanent damage to the respiratory tract. Recently, the incidence of NTM infections has been increasing in patients with bronchiectasis. Multiple small nodules, along with the occasional appearance of 1 or more cavities combined with diffuse bronchiectasis, are reported to be the typical high-resolution computed tomography (HRCT) findings in NTM-LD associated with bronchiectasis.^[5]^ In patients with characteristic HRCT findings, 34% to 50% of patients have an active NTM-LD.^[5]^

Prior studies reported that a variety of environmental and host factors have been associated with the development of NTM-LD in patients with bronchiectasis.^[5,6]^ Particularly, female patients, tall statured patients, and patients with low body weights are more susceptible to NTM-LD.^[7]^ Recent studies demonstrated that the susceptibility of a patient with a thin body to NTM-LD is related to dysregulation of hormones produced by fat tissue.^[8,9]^ Notably, alterations in adipokine levels, implicated in the regulation of immune responses, have been noted to influence the susceptibility to NTM-LD.

We hypothesized that individuals with a low-fat mass are susceptible to the development of NTM-LD. We expect that fat mass would be lower in patients with NTM-LD at the time of initial diagnosis regardless of the severity of bronchiectasis. Thus, we investigated the association between body composition measured by bioimpedance analysis (BIA), and concurrent NTM-LD in bronchiectasis patients. We also aimed to understand the effects of body composition on the progression of NTM-LD in patients with bronchiectasis.

## 2 Methods

### 2.1 Study population and design

The retrospective study was composed of consecutive nonhospitalized patients with noncystic fibrosis bronchiectasis referred to a tertiary care university hospital in Korea between January 2013 and January 2016. Axial images of HRCT scans were used to diagnose bronchiectasis. The specific criteria for bronchiectasis were the following: the internal diameter of the bronchus is larger than that of its accompanying vessel or the bronchus fails to taper in the periphery of the chest.^[10]^ The severity of bronchiectasis was measured using the bronchiectasis severity index score.^[11]^ Microbiologic examinations for NTM infection and BIA tests for body composition were performed in these patients. The following patients were excluded: patients with active tuberculosis or predominantly tuberculosis-destroyed lung, patients with traction bronchiectasis due to severe emphysema or fibrosis, patients previously treated for NTM lung disease, patients with fibrocavitary lung disease, patients with human immunodeficiency virus infection, immunosuppressant therapy, or malignancy. We divided the patients into 2 groups according to the presence of NTM-LD that fulfilled the diagnostic criteria as provided in the 2007 American Thoracic Society/Infectious Diseases Society of America guidelines for NTM infection.^[12]^ Progression of NTM-LD was defined as the initiation of anti-NTM treatment by the duty physician based on the clinical presentation and follow-up radiographic aggravation. The Institutional Review Board of Seoul National University Bundang Hospital, Seoul, South Korea, approved the study (approval no. B-1912-582-106) and waived the need for informed consent because no patients were at risk since the study was retrospective in nature.

### 2.2 Data collection

The baseline data at the time of diagnosis of bronchiectasis including respiratory symptoms and patient history, anthropometric parameters (weight, height, BMI), sputum culture, laboratory findings, pulmonary function test results, and chest CT findings were collected from hospital electrical medical record system.

### 2.3 Microbiological evaluation

A minimum of 3 sputum cultures for NTM was examined at diagnosis for every patient with a productive cough. At least 1 bronchoscopy for microbiological examination was performed in patients without productive cough but with other respiratory symptoms. Microbiological examinations were performed on sputum, bronchial aspiration, or bronchoalveolar lavage during the stable state. Acid-fast bacilli smears and cultures were done using standard methods.

### 2.4 Measurement of body composition

The assessment of body composition was evaluated either during the time of initial or subsequent clinic visits using BIA, which was performed using the InBody 720 device (Biospace Co, Seoul, South Korea). BIA provides resistance and reactance measured at different frequency currents, providing estimations of the body fat mass, protein mass, and skeletal muscle mass.^[13,14]^ The skeletal muscle mass index and FMI were calculated by dividing the subject's muscle and fat masses in kilograms by the square of the subject's height in meters. Body FMI was analyzed both as continuous variables and categorical variables, stratified by their quartile values within the patients with NTM-LD.

### 2.5 Statistical analyses

Comparisons between the 2 groups were performed by the Student *t* test or the Mann–Whitney *U* test for numerical data, and the χ2 test or Fisher exact test for categorical data. To identify the risk factors for the development of NTM-LD, we initially conducted a univariate analysis, and statistically significant variables (*P* < .05) in the univariate analysis were included in the multivariate regression analysis with forward conditional elimination of data. Data are presented as odds ratios (ORs) with 95% confidence intervals (CIs).

Cumulative disease progression was estimated using the Kaplan–Meier method. A cox-proportional hazard regression model was used to find predictors of disease progression. Covariates with a *P* value < .2 were used in multivariate analyses. Multivariate analyses were constructed using the stepwise backward elimination method. All analyses were performed using the R software (version 3.3.2; http://www.R-project.org), and *P* values of < .05 were considered statistically significant.

## 3 Results

**Figure 1:**
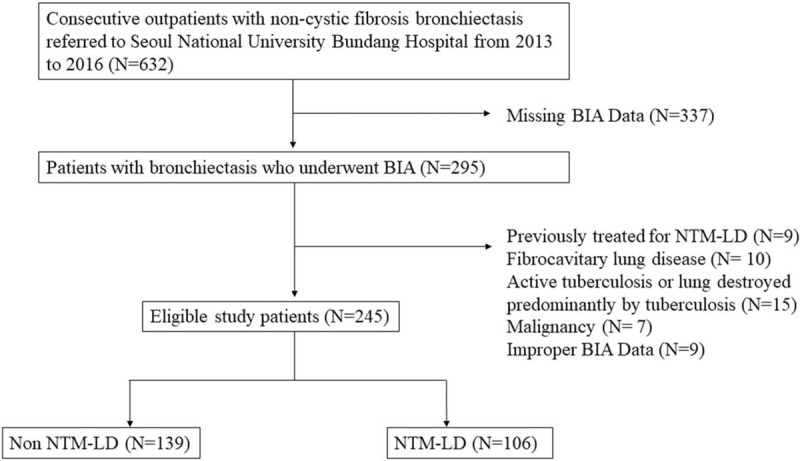
Flow chart showing the selection process of patients with bronchiectasis.

A total of 245 patients diagnosed with noncystic fibrosis bronchiectasis satisfied the inclusion criteria and were enrolled in the study (Fig. 1). Patients were grouped as those with NTM (n = 106, 43.3%) or without NTM LD (n = 139, 56.7%). The characteristics of the patients are summarized in Table 1. The mean (± standard deviation) age of non-NTM and NTM group patients was 59.55 (± 9.81) and 59.67 (± 10.01) years, respectively. Patients with NTM-LD were more likely to be females (24.8% vs 37.1%) and more likely to have a higher FEV1 (89.41 ± 22.44 vs 80.04 ± 24.54) when compared with the non-NTM group. There was no significant difference in the presence of comorbidities such as COPD, or malignancy between groups. There was also no significant difference in the bronchiectasis severity index between groups.

**Table 1:** Baseline characteristics.

	Total (N = 245)	Non-NTM (N = 139)	NTM (N = 106)	*P* value
Age		59.55 ± 9.81	59.67 ± 10.01	.921
Sex (male, %)	81 (31.64%)	53 (37.06%)	28 (24.78%)	.05
Comorbidities, n (%)				
Malignancy	20 (7.84%)	10 (7.04%)	10 (8.85%)	.765
Diabetes	28 (10.98%)	14 (9.86%)	14 (12.39%)	.660
COPD	27 (10.59%)	19 (13.38%)	8 (7.08%)	.156
Asthma	76 (29.80%)	51 (35.92%)	25 (22.12%)	.024
Past medical history, n (%)				
Tuberculosis	76 (29.80%)	39 (27.46%)	37 (32.74%)	.437
Measle	91 (37.45%)	55 (39.29%)	36 (34.95%)	.578
Pertussis	29 (12.24%)	21 (15.67%)	8 (7.77%)	.101
Smoking, n (%)				.037
Never	189 (73.83%)	99 (69.23%)	90 (79.65%)	
Former	7 (2.73%)	7 (4.90%)	0 (0.0%)	
Current	58 (22.66%)	35 (24.48%)	23 (20.35%)	
Symptoms at initial visit				
Cough	91 (35.69%)	56 (39.44%)	35 (30.97%)	.204
Sputum	71 (27.84%)	40 (28.17%)	31 (27.43%)	1.000
Hemoptysis	91 (35.55%)	53 (37.06%)	38 (33.63%)	.661
Weight loss	48 (19.20%)	25 (17.99%)	23 (20.72%)	.701
Pulmonary function				
FVC (L)	2.64 ± 0.73	2.64 ± 0.72	2.65 ± 0.74	.888
FVC (%)	86.89 ± 18.06	85.38 ± 17.40	88.81 ± 18.79	.180
FEV1 (L)	1.81 ± 0.58	1.75 ± 0.58	1.88 ± 0.59	.118
FEV1 (%)	84.15 ± 24.04	80.04 ± 24.54	89.41 ± 22.44	.006
Bronchiectasis severity index	3.94 ± 2.15	4.16 ± 2.13	3.66 ± 2.15	.110
Protein (g/dL)	7.2 ± 0.5	7.2 ± 0.5	7.2 ± 0.4	.475
Albumin (g/dL)	4.2 ± 0.3	4.1 ± 0.4	4.2 ± 0.3	.181
Cholesterol (mg/dL)	176.9 ± 33.2	176.8 ± 33.1	177.1 ± 33.4	.948

Values expressed as mean ± standard deviation, or n (%). COPD = chronic obstructive pulmonary disease, FEV1 = forced expiratory volume in 1 second, FVC = forced vital capacity, NTM = nontuberculous mycobacterial lung disease.

### 3.1 Body composition of bronchiectasis patients with or without NTM-LD

Table 2 shows the body composition of patients with bronchiectasis with or without NTM. The mean body weight was 54.16 kg (±8.99) and 58.20 kg (±8.84) in the NTM and non-NTM group, respectively (*P* < .001). The mean BMI of patients with NTM-LD was significantly lower than that of those without NTM (21.20 ± 2.59 vs 22.67 ± 3.04 kg/m^2^, *P* < .001). Particularly, the NTM-LD group had less fat mass than the non-NTM group (14.23 ± 5.79 vs 16.19 ± 6.60 kg, *P* = .013). Additionally, the FMI in the NTM group was lower than that in the non-NTM group (5.57 ± 2.27 vs 6.79 ± 2.70 kg/m^2^, *P* < .001). However, in contrast with fat composition, there were no significant differences in protein and skeletal muscle indices between those with and without NTM.

**Table 2:** Body composition at study enrollment.

	Total (N = 245)	Non-NTM (N = 139)	NTM (N = 106)	*P*-value
Height (cm)	159.95 ± 7.99	160.23 ± 7.90	159.59 ± 8.12	.530
Weight (kg)	56.41 ± 9.12	58.20 ± 8.84	54.16 ± 8.99	<.001
BMI (kg/m^2^)	22.02 ± 2.93	22.67 ± 3.04	21.20 ± 2.59	<.001
Total body water (kg)	30.33 ± 5.80	31.04 ± 6.12	29.42 ± 5.26	.026
Protein (kg)	7.93 ± 1.61	8.06 ± 1.73	7.78 ± 1.44	.157
Mineral (kg)	2.91 ± 1.54	3.10 ± 1.80	2.67 ± 1.10	.020
Fat (kg)	15.32 ± 6.32	16.19 ± 6.60	14.23 ± 5.79	.013
Soft lean mass (kg)	38.55 ± 7.69	39.24 ± 8.32	37.67 ± 6.74	.098
Skeletal muscle mass (kg)	21.94 ± 4.87	22.32 ± 5.23	21.45 ± 4.36	.147
Skeletal muscle index (kg/m^2^)	6.87 ± 3.85	7.20 ± 5.04	6.45 ± 1.01	.088
Percent body fat (%)	26.78 ± 9.35	27.45 ± 9.90	25.93 ± 8.58	.196
Visceral fat (%)	68.16 ± 26.21	71.87 ± 27.17	63.47 ± 24.25	.003
Fat mass index (kg/m^2^)	6.27 ± 2.59	6.79 ± 2.70	5.57 ± 2.27	<.001
Phase angle	4.70 ± 0.82	4.76 ± 0.87	4.60 ± 0.73	.457

Values expressed as mean ± standard deviation, or n (%). BMI = body mass index, NTM = nontuberculous mycobacterial lung disease.

### 3.2 Predictive factors of the presence of NTM lung disease

The logistic regression model for NTM-LD in patients with bronchiectasis is shown in Table 3. In univariate analysis, the probability of NTM-LD in bronchiectasis patients was significantly higher in females, patients with lower BMIs, and patients with a lower fat mass, although there was no association with protein and skeletal muscle mass. Female sex and lower FMI were found to be the only independent risk factors of NTM-LD after adjusting for age, bronchiectasis severity index, and BMI (odds ratio 3.86 [1.99–7.78]; 0.72 [0.63–0.82], *P* < .001, respectively).

**Table 3:** Risk factors of NTM infection in patients with bronchiectasis.

	Univariate		Multivariate	
	OR (95% CI)	*P* value	OR (95% CI)	*P* value
Age	1.00 (0.97-1.02)	.8305		
Sex	1.90 (1.09-3.35)	.0250	3.86 (1.99–7.78)	.0001
BMI	0.77 (0.69–0.86)	.0001		
Protein	0.86 (0.73–1.02)	.0857		
Fat	0.91 (0.87–0.96)	.0001		
Skeletal muscle mass	0.95 (0.90–1.00)	.0785		
Skeletal muscle mass index	1.00 (0.82–1.20)	.9890		
Visceral fat	0.98 (0.97–0.99)	.0003		
Fat mass index	0.82 (0.73–0.91)	.0004	0.72 (0.63–0.82)	<.0001
Bronchiectasis severity index	0.90 (0.79–1.01)	.0739	0.89 (0.78–1.02)	.1056

BMI = body mass index, CI = confidence interval, NTM = nontuberculous mycobacterial lung disease, OR = odds ratio.

### 3.3 Subgroup analysis according to the fat mass index

**Figure 2:**
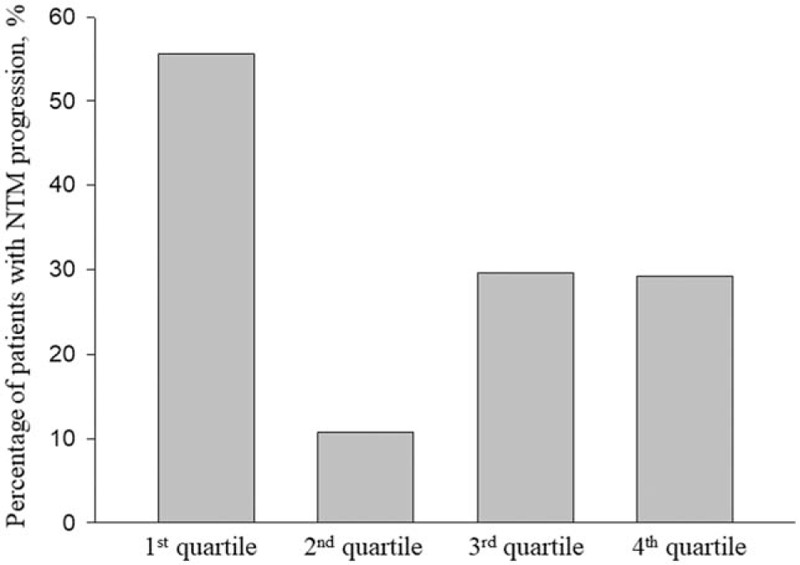
Percentage of NTM progression according to fat mass index quartiles. NTM = nontuberculous mycobacterial lung disease.

**Figure 3:**
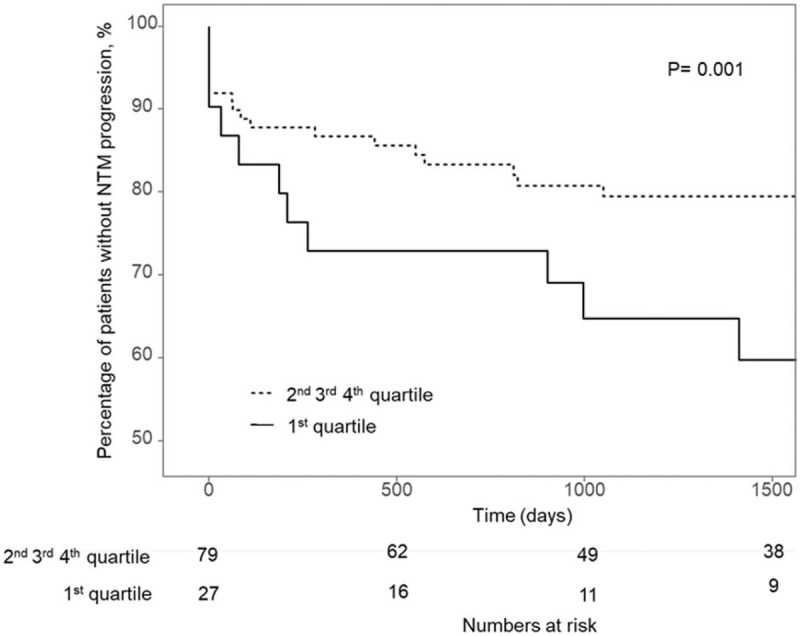
Disease progression among patients with nontuberculous mycobacterial infection and bronchiectasis NTM nontuberculous mycobacterial lung disease.

Patients with NTM-LD were divided into 4 quartiles according to FMI, and the body composition and clinical outcomes of each quartile were compared (Table 4 and Fig. 1). BMI was significantly lower in patients in the first quartile of FMI. However, no significant difference was found in skeletal muscle mass index between quartiles. A higher incidence of NTM-LD progression was observed in the lowest quartile than in the other upper quartiles (Figs. 2 and 3). Moreover, the first quartile of FMI was found to be an independent predictor of NTM progression in multivariate cox regression analyses (Table 5, hazard ratio, 2.90 (1.46–5.77), *P* = .002).

**Table 4:** Body morphotype and Mycobacterial species according to fat mass index quartiles.

	First quartile (N = 27)	Second quartile (N = 28)	Third quartile (N = 27)	Fourth quartile (N = 24)	*P* value
Age	63.3 ± 10.0	56.3 ± 10.3	56.8 ± 9.6	62.6 ± 9.8	.824
Bronchiectasis severity index	4.6 ± 2.0	3.2 ± 2.2	3.3 ± 1.9	3.7 ± 2.4	.155
BMI	19.5 ± 1.9	20.0 ± 1.8	21.0 ± 1.8	23.7 ± 2.4	.000
Skeletal muscle index (kg/m^2^)	6.9 ± 1.3	6.5 ± 0.9	6.1 ± 1.0	6.5 ± 0.9	.073
Mycobacterial species					.106
*Mycobacterium avium* complex	20 (83.3)	19 (67.9)	19 (73.1)	14 (58.4)	
*M abscessus* complex	3 (12.5)	4 (14.3)	4 (15.4)	8 (33.3)	
Others	1 (4.2)	5 (17.9)	3 (11.5)	2 (8.3)	

Values expressed as mean ± standard deviation, or n (%). BMI = body mass index, NTM = nontuberculous mycobacterial lung disease, Others = *Mycobacterium avium* complex and *M abscessus* complex.

**Table 5:** Predictors of NTM lung disease progression: a multivariate analysis.

	Univariate		Multivariate	
	HR (95% CI)	*P* value	HR (95% CI)	*P* value
age	1.02 (0.99–1.06)	.149	1.01 (0.97–1.06)	.497
Sex	0.58 (0.28–1.20)	.142	1.19 (0.48–2.93)	.703
BMI	1.01 (0.88–1.16)	.866		
Skeletal muscle index	1.00 (0.70–1.41)	.985		
Bronchiectasis severity index	1.13 (0.97–1.33)	.122	1.03 (0.83–1.28)	.791
Fat mass index (vs second, third, and fourth quartiles)				
First quartile group	2.88 (1.45–5.73)	.003	2.72 (1.19–6.26)	.018

BMI = body mass index, CI = confidence interval, HR = hazard ratio, NTM = nontuberculous mycobacterial lung disease.

## 4 Discussion

The present study demonstrates that in patients with noncystic bronchiectasis, NTM-LD occurred dominantly in females and was significantly associated with lower BMI and body fat. Patients with NTM-LD also had reduced percent visceral fat and lower FMI, and lower FMI was independently associated with a greater risk of having NTM-LD. Our study also showed that the lowest quartile of FMI was found to be an independent predictor of NTM-LD progression. However, body protein and skeletal muscle index were not different between patients with and without NTM-LD.

A prior study demonstrated that nodular bronchiectatic type NTM-LD is often observed in thin middle-aged and older women, and a slender body morphotype has been reported as a prognostic factor.^[8]^ Several studies have also consistently shown that in otherwise healthy subjects, low BMI and the female sex were risk factors of NTM-LD.^[7,8]^ While BMI is the most commonly used parameter to characterize body morphotype, BMI can neither distinguish nor quantify muscle mass and fat mass.^[15]^ To overcome this limitation, the BIA method was used in the current study, which can estimate the exact component of body compositions.

In our study, aside from protein or muscle mass, lack of fat mass showed a strong association with NTM-LD and its progression. Fat mass was found to help preserve the immune response to various infectious diseases.^[16]^ In an experimental model of sepsis caused by cecal ligation and puncture in C57BL/6 mice, animals prefed with a high-fat diet to induce obesity showed markedly enhanced survival.^[17]^ Clinical studies have also confirmed the protective effects of body fat against various infectious diseases including tuberculosis, *Streptococcus pneumonia*, and HIV infection.^[18–20]^

Chan and Iseman^[8]^ suggested that the susceptibility of a patient with a thin body to NTM-LD is related to hormones produced by adipose tissue. Altered hormone levels in thin individuals may modulate the host response in terms of cytokine and chemokine expression and, in turn, contribute to predisposition to NTM infections. Tasaka et al^[21]^ found that patients with NTM infection had lower serum leptin and higher adiponectin levels than healthy control participants matched for age, gender, and BMI. Lower leptin levels account for reduced lymphopoiesis and impaired production of host protective cytokines, whereas adiponectin is an immunosuppressive adipokine that induces anti-inflammatory mediators, leading to increased susceptibility to infections in increased levels of adiponectin.^[9]^ Similarly, Kartalija et al^[22]^ revealed that patients with NTM lung disease had a lower body fat calculated by the Durnin/Womersley caliper method and that there is regulatory dysfunction of leptin and adiponectin in these subjects.

Consistent with our results, 103 patients with NTM-LD had a markedly reduced visceral fat area assessed by abdominal CT, compared with that of generally healthy adults.^[23]^ Kim et al^[24]^ also reported similar results when analyzing data from participants in a prospectively recruited, observational NTM cohort. They found that a lower abdominal fat assessed by BIA, and the presence of cavity on CT scans were associated with the progression of NTM-LD. This study was mainly conducted in patients with underlying lung disease such as tuberculosis-associated destroyed lung, which may confound some of the findings and interpretations. However, patients with tuberculosis-destroyed lung or fibrocavitary type of NTM infection, in which underlying pulmonary disease was common, were excluded from our study. Furthermore, patients with bronchiectasis but without NTM infection were used as controls rather than healthy volunteers.

There are a few limitations to this study. First, this was a single-center study; hence, the data should be confirmed with larger samples and in studies conducted in multiple centers. Second, the inherent limitations with any retrospective design with a relatively small number of male patients largely limit its generalizability. However, all epidemiologic studies consistently show more that more women than men had bronchiectasis and NTM-LD which has been observed more frequently in women among patients with bronchiectasis. Additionally, we were unable to draw suitable conclusions about the causal relationship and the exact mechanisms contributing to the development of NTM-LD in patients with a low-fat mass. Nevertheless, our results showed no differences in fat mass according to the severity of bronchiectasis. Furthermore, we observed that patients with NTM disease progression showed a lower FMI than those without progression. To prove a causal relationship, further studies on the correlation between low fat intake and NTM infection are needed. Finally, fat mass measurements were not performed by computed tomography, which is currently accepted as the ideal standard for the evaluation of muscle mass and other body components.^[25]^ However, BIA measurement of body composition has previously been validated, and it has been widely used to assess different body compositions in patients with various lung diseases, due to its high clinical accessibility.^[26]^

In conclusion, our results suggest that abnormal body composition is an important modifiable risk factor in patients with bronchiectasis, particularly with concomitant NTM-LD. BIA provides a simple, inexpensive, and reliable estimate for the quantification of different body components. Therefore, early nutritional support in patients with a low-fat mass, as measured by BIA, may play a role in optimizing treatment outcomes.

## Author contributions

**Conceptualization:** Sung Yoon Lim, Jong Sun Park, Young-Jae Cho.

**Data curation:** Sung Yoon Lim, Yeon Joo Lee, Jong Sun Park.

**Formal analysis:** Sung Yoon Lim.

**Investigation:** Ho Il Yoon.

**Methodology:** Sung Yoon Lim, Jong Sun Park.

**Supervision:** Ho Il Yoon, Choon-Taek Lee, Jaeho Lee.

**Validation:** Yeon Joo Lee, Young-Jae Cho.

**Writing – original draft:** Sung Yoon Lim.

**Writing – review & editing:** Jaeho Lee.
